# Natural Populations of Shipworm Larvae Are Attracted to Wood by Waterborne Chemical Cues

**DOI:** 10.1371/journal.pone.0124950

**Published:** 2015-05-13

**Authors:** Gunilla B. Toth, Ann I. Larsson, Per R. Jonsson, Christin Appelqvist

**Affiliations:** University of Gothenburg, Department of Biological and Environmental Sciences-Tjärnö, Strömstad, Sweden; University of New South Wales, AUSTRALIA

## Abstract

The life cycle of many sessile marine invertebrates includes a dispersive planktonic larval stage whose ability to find a suitable habitat in which to settle and transform into benthic adults is crucial to maximize fitness. To facilitate this process, invertebrate larvae commonly respond to habitat-related chemical cues to guide the search for an appropriate environment. Furthermore, small-scale hydrodynamic conditions affect dispersal of chemical cues, as well as swimming behavior of invertebrate larvae and encounter with potential habitats. Shipworms within the family Teredinidae are dependent on terrestrially derived wood in order to complete their life cycle, but very little is known about the cues and processes that promote settlement. We investigated the potential for remote detection of settling substrate via waterborne chemical cues in teredinid larvae through a combination of empirical field and laboratory flume experiments. Natural populations of teredinid larvae were significantly more abundant close to wooden structures enclosed in plankton net compared to empty control nets, clearly showing that shipworm larvae can sense and respond to chemical cues associated with suitable settling substrate in the field. However, the flume experiments, using ecologically relevant flow velocities, showed that the boundary layer around experimental wooden panels was thin and that the mean flow velocity exceeded larval swimming velocity approximately 5 mm (≈ 25 larval body lengths) from the panel surface. Therefore, we conclude that the scope for remote detection of waterborne cues is limited and that the likely explanation for the higher abundance of shipworm larvae associated with the wooden panels in the field is a response to a cue during or after attachment on, or very near, the substrate. Waterborne cues probably guide the larva in its decision to remain attached and settle, or to detach and continue swimming and drifting until the next encounter with a solid substrate.

## Introduction

The life cycle of many marine invertebrates includes a sessile adult phase and a planktonic larval phase that may remain suspended in the water column for minutes to months [1,2]. A successful transition from the planktonic larval phase to the sessile adult phase includes settling and metamorphosis and requires a suitable habitat. Settling close to abundant food sources, away from competitors and predators, but close to conspecifics is crucial for many sessile benthic invertebrates and will greatly affect fitness (growth, survival, and/or reproduction) [3–5]. A major challenge for invertebrate larvae, many of which are small and unable to swim against the current velocities dominating in coastal areas, is successful location of suitable habitats and settling substrate. In the first half of the 20th century, the deposition of invertebrate larvae and larval settlement was thought to be an entirely random process [6]. However, during the last 50 years, the view of larval settlement has changed from larvae as passive particles transported by currents, to organisms possessing highly complex sensory organs capable of sensing, recognizing and responding to different habitat-related cues [7–11].

Settlement and metamorphosis of benthic invertebrate larvae are commonly influenced by hydrodynamic, chemical, visual, tactile, and/or acoustic cues and different types of cues have different ranges of effectiveness. Acoustic and hydrodynamic cues (e.g. sound characteristics and, e.g. turbulent shear typical of wave swept shores) are considered large-scale habitat-cues that signal larvae that they are in the right habitat [12,13], while visual, tactile, and chemical cues, as well as small-scale hydrodynamics are considered effective at short ranges [14,15]. Many invertebrate larvae use a combination of cues to ensure successful location of suitable settling habitats [12].

Apart from functioning as habitat cues, hydrodynamic turbulence and flow advection affect the dispersal of chemical cues in the water column, as well as the trajectories and swimming behavior of invertebrate larvae [16]. Under realistic flow conditions, chemical cues that are released from submerged surfaces form filaments that swirl around in cue free water [17]. Therefore, rather than encountering a diffuse concentration gradient as has previously been hypothesized [18], larvae move in and out of filaments with different cue concentrations as they swim or sink through the water column [17]. Experiments done in the laboratory and field with unidirectional flow conditions have shown that turbulent flow in the boundary layer affects the delivery of the larvae to the substratum [19,20]. However, ignoring temporal and spatial variation in fine-scale flow and cue concentrations can overestimate the rate of transport of the larvae to the substratum [17]. Furthermore, the swimming behavior of larvae in realistic water flow conditions may be altered [21–27], and should be taken into account when modeling the contact-rate of larvae to settling substrate [17]. Therefore, to estimate the potential for remote detection of suitable settling substrate for marine invertebrate larvae, the dispersal of chemical cues and the trajectories of the larvae have to be measured and/or modeled under realistic flow conditions [16].

Shipworms (Family Teredinidae) belong to a diverse group of highly specialized marine bivalves that use terrestrially derived wood as both habitat and food [28,29]. The adult shipworms use their modified shells as tools to bore into wooden substrate. Burrows are initiated when planktonic larvae infest the wood, and are expanded as the individuals grow. The wooden substrate is steadily decomposed through the feeding activity and growth of the shipworms, and therefore shipworms can cause massive damage to man-made wooden structures [30]. The reproductive strategy of the majority of shipworm species is oviparous, releasing a large number of gametes or fertilized eggs into the water for planktotrophic development of 20 days or more [31,32]. However, a few genera are larviparous and brood fertilized eggs in modified gill chambers to straight-hinge veliger or pediveliger larval stage before release into the plankton [32,33]. These larvae are more developed at the time of release and are able to settle within hours (pediveliger larvae), or after about 15 days (veliger larvae) of additional planktotrophic development. Irrespective of reproductive strategy, the larvae need to localize a suitable woody substrate in order to complete the life cycle. To our knowledge, no previous study has explicitly tested whether natural populations of shipworm larvae can remotely detect settling substrate through waterborne cues under ecologically realistic conditions.

The overall aim of this study was to investigate the potential for remote detection of suitable settling substrate in temperate shipworms. Firstly, we investigated if shipworm larvae are able to sense chemical cues released from wooden structures. This was done by collecting larvae around submerged wooden panels enclosed in plankton net in a field setting. Secondly, instantaneous flow velocities, as well as spreading of cues around wooden panels were measured in flume experiments using ecologically relevant hydrodynamic conditions. Together with estimations of the swimming speed of shipworm larvae in culture, these data were used to calculate theoretical distances from which shipworm larvae should be able to respond to cues released from submerged wooden substrates in the field.

## Materials and Methods

### Study site and organisms

The experiments were performed outside the Sven Lovén Center for Marine Sciences—Tjärnö (SLCT, 58.88° N, 11.15° E) on the Swedish west coast. No specific permissions were required to sample organisms at these locations and the study did not involve any endangered or protected species. The study area is characterized by an archipelago with many small islands and islets. The tidal range is only about 20 cm, but the mean water level can fluctuate a few meters depending on the prevailing air pressure and wind direction. The coast is dominated by the Baltic surface current consisting of outflowing water from the Baltic Sea following the Swedish west coast due to the earth’s rotation. In the northern part of the west coast, the northward-moving Jutland coastal current moves around northern Denmark towards the Swedish coast. The Jutland coastal current has higher salinity and therefore flows under the Baltic current. Current speed in the Baltic surface current is generally 20 to 100 cm s^-1^, although this is reduced close to shore or within shallow parts of the archipelago.

Two species of teredinid shipworms (*Teredo navalis* and *Psiloteredo megotara*) are found at relatively shallow depth (0–20 m) along the Swedish west coast. *T*. *navalis* is found in high numbers in the study area, while *P*. *megotara* is less abundant [34]. *T*. *navalis* is a protandric hermaphrodite with internal fertilization [33,35]. The offspring are brooded until larvae reach the straight-hinge veliger stage (75 x 80 μm), after which larvae are released to the plankton [36]. The straight-hinge veligers develop for a further 2–3 weeks into pediveliger larvae (200–240 μm), after which they settle onto wooden substrates [36]. In contrast to *T*. *navalis*, *P*. *megotara* have external fertilization and a free-swimming period of about 4 weeks [37].

### Field experiments

#### Detection of chemical cues by shipworm larvae in the field

**Fig 1:**
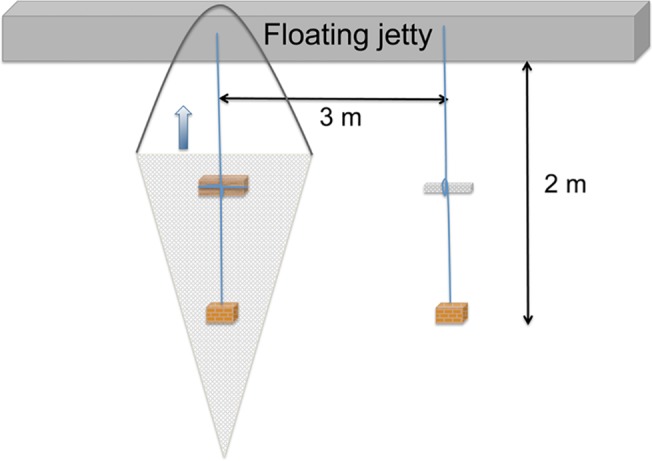
Field experiment. Experimental set up used to study detection of waterborne chemical cues by shipworm larvae in the field. Net bags with and without wooden panels were submerged at 1 m depth from a floating jetty. A vertical tow with a hoop-net (90 μm, illustrated by a grey triangle) was used to filter the water around the net bags. In each tow, the hoop-net including the net bag and its line was lifted to the surface.

In order to investigate if shipworm larvae are able to detect suitable substrate via waterborne chemical cues under field conditions, 8 Norway spruce (*Picea abies*) panels (120 x 60 x 30 mm) were deployed at 1 m depth outside the SLCT in August 2011. The panels were placed in bags made from plankton net (50 μm) and attached to lines weighted down by brick stones (Fig 1). Control net bags, with similar shape but without wooden panels, were also prepared and weighted in the same way. Net bags with and without wooden panels were placed randomly with 3 m intervals from a dock. At two occasions (after 8 and 16 days), vertical tows were made using a hoop-net (90 μm) (Fig 1). Through this procedure, 192 L of water around each net bag was filtered. The hoop-net was thoroughly rinsed and collected larvae were fixed in ethanol (96%). The procedure was repeated in a random order for all net bags. Teredinid pediveliger larvae in the plankton samples were identified through their characteristic oval shape (i.e. greater height than length), yellowish color, and lack of "eyespot", which distinguish them from other bivalve larvae [38,39].

Data on the number of shipworm larvae were statistically analyzed using an orthogonal 2-way analysis of variance (ANOVA) using Treatment and Tow as a fixed 2-level factors. Prior to statistical analysis, data were tested for homogeneity of variances using Cochran´s test. Statistically significant differences between mean values were statistically analyzed using the Student-Newman-Keuls (SNK) multiple-comparisons test.

#### Measurement of water flow in the field

The hydrodynamic conditions around the floating dock where the net bags with and without wooden panels were submerged were monitored using an acoustic doppler current profiler (ADCP, Nortek AS). The ADCP was placed at the bottom a few meters from the floating dock (water depth 3.5 m) and measured the flow velocity every hour during 3 weeks in layers from 1.5 m above the sea-floor to the water surface. A cell size of 0.3 m was used and the data from the cell measuring at the depth were panels had been deployed was extracted.

### Laboratory experiments

#### Estimation of larval swimming speed in culture

Scots pine (*Pinus sylvestris*) panels were submerged during August and September 2010 outside SLCT. After collection, any biofouling on the surface of the panels was removed and the panels were kept in filtered (5 μm) flow-through seawater aquaria (17°C, 28 PSU) in the lab. Straight-hinge veliger larvae released in the aquaria were collected every day on 45 μm nylon sieve.

Larval swimming speed was measured by video recording newly released cultured veliger larvae (75 x 80 μm) in a Petri dish. The videos were played-back in slow motion and 10 larvae with rapid movement were measured in order to estimate the maximum speed. Swimming speed was calculated as the distance in mm that larvae moved per s. Swimming speed increase with increasing larval size [36], and in order to estimate the swimming speed of 200 μm pediveliger larvae, a published correlation between larval size and swimming speed for veliger larvae of blue mussel (*Mytilus edulis*) was used [40].

#### Flume experiments

Flow patterns around wooden panels and transport of chemical substances released from these panels were studied in a laboratory flume. We used the same size and type of panels that were previously used in the field study. The 7 m long and 0.5 m wide recirculating flume (for further description see [41]) was filled to a water depth of 20 cm and the seawater (salinity 33.5 ‰) was maintained at 18°C. An acoustic doppler velocimeter (ADV, Nortek AS) measured the free stream velocity at 10 cm above the flume floor. Measurements on wooden panels were performed in average free stream velocities of 1, 3 and 5 cm s^-1^ (± 0.2 cm s^-1^) based on field data collected with the ADCP. The wooden panels were placed horizontally in the working section situated 5 m from the flume entrance with the smallest cross section area (the end grain) in the flow direction. The 30 mm thick panels were mounted with the bottom of the panels 2 cm above the flume floor using two screws. Studies of flow patterns around the panels were done using particle image velocimetry (PIV) and laser induced fluorescence (LIF) was employed to study release and transport of water-soluble chemical substances from the wooden panels. For these 2 techniques we used a system from LaVision with a 1600×1200 pixel camera (Imager Pro X) and a double pulsed Nd:YAG laser (Litron, 30 mJ at 532 nm). The camera with a 24-mm Nikkor lens recorded images through the transparent sidewall of the flume covering an area of 16×12 cm where the panels were placed. The laser produced a 1 mm thick vertical light sheet parallel to the flow and in the center of the top-side of the panels where hence PIV and LIF recordings were taken.

For LIF measurements, wooden panels were soaked in seawater with 2 mg l^-1^ Rhodamine 6G for two days. This fluorescent dye mimics any water-soluble chemicals that might act as attractive cues. After mounting the panel in the flume it was left for 1 hour at the flow speed of 1 cm s^-1^. This time allowed the fluorescent dye to start diffusing out of the panel and a diffusive boundary layer to form. LIF measurements were made at 10 Hz during 5 minutes (3000 recordings) using a 540 nm cutoff filter allowing the fluorescent light, but not the laser light, to pass. The recordings were made in the order 1, 3 and last 5 cm s^-1^ because the diffusive boundary layer was expected to become eroded (thinner) with increasing flow speed. Recorded images were corrected for camera background intensity and laser sheet inhomogeneities using the DaVis 8.2.0 software (LaVision). A concentration calibration was not applied since we did not use the LIF recordings for any quantitative measurements, but to study flow effects on thickness of the diffusive boundary layer and distribution of dye filaments around wooden panels.

For PIV recordings the wooden panel was painted black to minimize reflections from the laser. The water in the flume was seeded with 10 μm hollow glass spheres (Dantec Dynamics) and recordings were made in double frame mode at 10 Hz for 2 minutes (1200 recordings) at each of the three free stream velocities. Images were analyzed with the DaVis 8.2.0 software (LaVision) using the multipass option with a final interrogation window size of 12×12 pixels and with 75% overlap between interrogation windows. This setup resulted in a resolution of 33 flow vectors per cm.

## Results

### Field experiments

**Fig 2:**
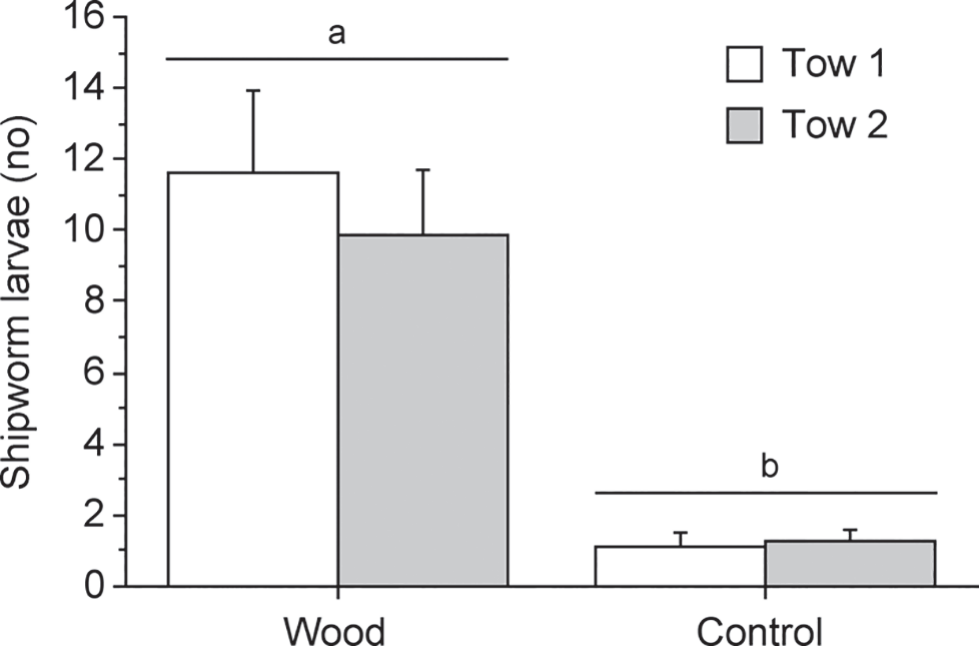
Field experiment. Number of shipworm larvae collected around wooden panels wrapped in plankton net (Wood) or plankton net only (Controls) at two occasions (Tow 1 and 2). Letters above bars indicate significant differences between mean values based on the Student-Newman-Keuls multiple comparisons test (SNK, *P* < 0.05). Error bars show + SEM (n = 8).

Data on the number of shipworm larvae in tows around empty plankton net (controls) or wooden panels wrapped in plankton net were log (x + 0.001) transformed prior to statistical analysis in order to fulfill assumptions of homogeneity of variances. Significantly more shipworm larvae were found around wooden panels compared to controls (ANOVA, *F*_1,28_ = 18.709, *P* = 0.0002). On average, 9 times more larvae were found in the water close to submerged wooden panels compared to empty plankton net (Fig 2). The difference in larval abundance was similar among occasions, as shown by the non-significant interaction between the factors Tow and Treatment (ANOVA, *F*_1,28_ = 0.021, *P* = 0.8852). Furthermore, there was no statistically significant difference in the number of shipworm larvae found between different occasions (ANOVA, *F*_1,28_ = 0.002, *P* = 0.9647).

**Fig 3:**
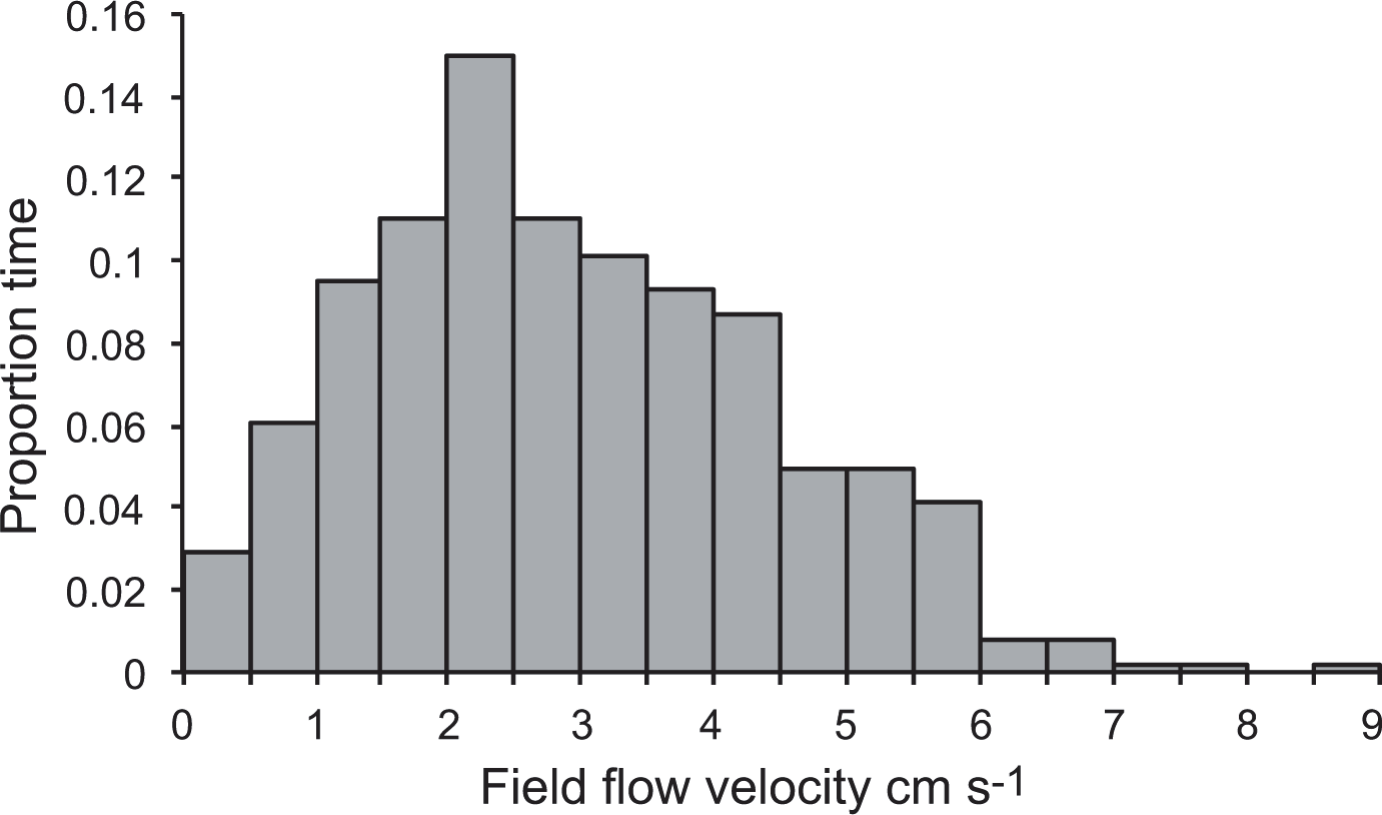
Field measurements. Proportional distribution of field flow velocities measured at 1 m depth outside the floating dock where wooden panels were deployed. Velocities were recorded every hour during 3 weeks using an acoustic doppler current profiler (ADCP).

The flow velocity around the dock where panels were deployed varied between < 0.5 cm s^-1^ to close to 9 cm s^-1^ with an average speed of 3.0 cm s^-1^ during the measurement period. The proportional distribution of flow velocities (Fig 3) show that the ambient flow speed was < 1 cm s^-1^ during 9% of the time, < 3 cm s^-1^ during 55% of the time and < 5 cm s^-1^ during 89% of the measurement period.

### Laboratory experiments

The maximum and average swimming speed of shipworm veliger larvae (75 x 80 μm) was 1.28 mm s^-1^ and 0.99 ± 0.08 (mean ± SEM) mm s^-1^ respectively. The average swimming speed of the shipworm larvae is comparable to similarly sized *Mytilus edulis* veliger larvae, and using a published linear regression between swimming speed and size [40], we estimated the swimming speed for pediveliger shipworm larvae to approximately 2 mm s^-1^.

**Fig 4:**
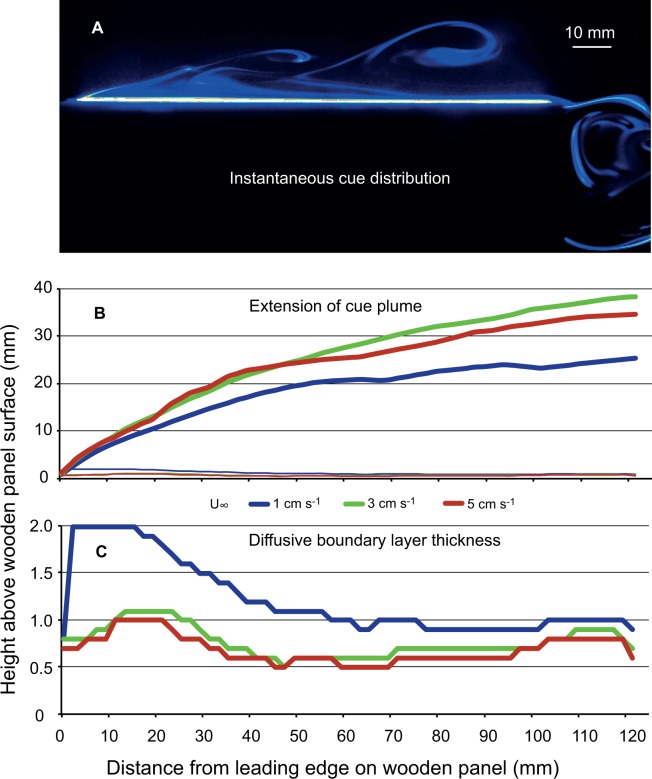
Laboratory flume experiment. Results from laser induced fluorescence (LIF) recordings on dye solution diffusing out of wooden panels. The fluorescent dye mimics any water soluble chemical that might act as attractive cues. A) Instantaneous cue distribution around the wooden panel at a free stream velocity of 1 cm s^-1^ with a high concentration diffusive boundary layer (DBL) on the panel surface and filaments of lower concentration transported from the DBL into the surrounding water. B) The maximum vertical distribution of cue filaments above the panel surface in the three tested free stream velocities; thin lines also show the DBL thickness for comparison. C) Average thickness of DBL as a function of free stream velocity and distance from the leading edge, i.e. upstream end of panel.

From instantaneous intensity images of the LIF recordings (Fig 4A) average and SD images were produced using the DaVis 8.2.0 software. The maximum vertical distribution of the dye filaments above the panel surface (extension of the cue "plume") in the different free stream velocities could most easily be derived from the SD images and are presented in Fig 4B. Below these lines approaching larvae could encounter attractive cue filaments. The diffusive boundary layer (DBL) on top of the wooden panels could clearly be distinguished on images from the LIF recordings (Fig 4A). The high concentration of Rhodamin dye in the DBL is shown as a white stripe of high fluorescing intensity on the panel surface. Measurements of the thickness of the DBL were done on average intensity images from the different free stream velocities. As expected, the DBL thickness decreased with free stream velocity although the difference between 3 and 5 cm s^-1^ is minor. The average DBL thickness also varied along the panel surface (Fig 4C), which can be explained by the flow pattern around the panels measured by PIV.

**Fig 5:**
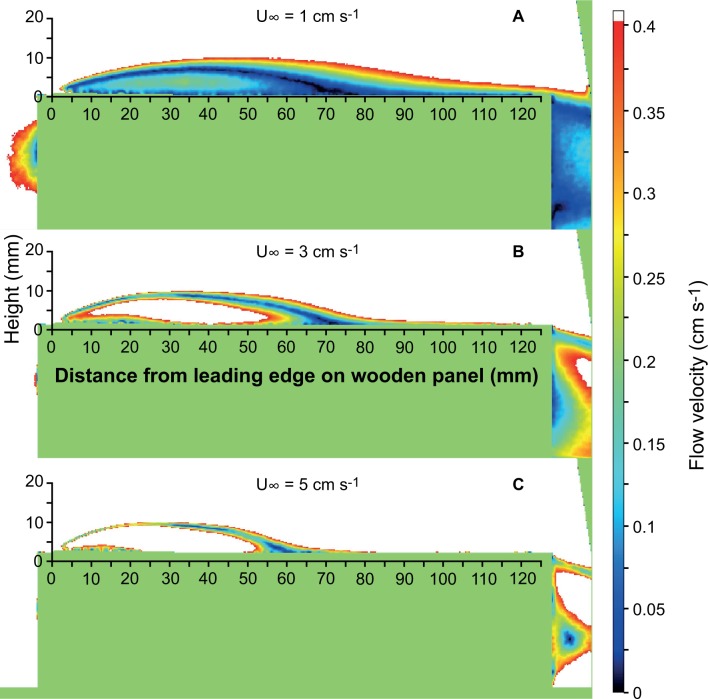
Laboratory flume experiment. Flow velocity fields around wooden panels measured with particle image velocimetry (PIV). Green color represents masked out areas that could not be analyzed and these are somewhat larger than the wooden panel. Coloured areas represent flow velocities below 0.4 cm s^-1^ i.e. twice the competent shipworm larva swimming speed. In white areas the flow velocity exceeds 0.4 cm s^-1^.

The average PIV vector fields show that there are 2 areas where the water is re-circulated resulting in flow speeds much lower than the free stream velocity. A recirculating cell is formed close to the surface just behind the leading edge (upstream end) of the panel and at the downstream end a wake is formed. In these recirculating areas, dye is accumulated (Fig 4A). Furthermore, downstream of the recirculating cell behind the leading edge the flow speed is reduced in the area close to the panel surface. Fig 5 shows the magnitude of the resulting flow velocities around the panel at the three free stream speeds tested. Velocities below 0.4 cm s^-1^, representing twice the competent shipworm larva swimming speed, are shown in color code whereas areas with flow velocities above 0.4 cm s^-1^ are shown in white. In the areas with velocities below 0.4 cm s^-1^, it can be expected that larvae sensing attractive cue filaments also can elicit a behavior that increase the possibility to encounter and attach to the wooden panel. In areas with flow velocities above 0.4 cm s^-1^, larval behavior most likely cannot affect the encounter probability since the larvae are quickly transported away.

## Discussion

Shipworms have attracted substantial attention from researchers for their unusual biology, morphology and ecology, not the least for their xylotrophic (wood feeding) behavior [28,31]. Infestation by shipworms may cause extensive economic and cultural problems through destruction of wooden maritime structures [30]. The distribution and abundance of shipworms is relatively well studied [31,32,42–46], but the process that allows shipworm larvae to find suitable settling substrate is still largely unknown. Shipworm recruitment is commonly studied by submerging wooden test panels in the sea [34,45,47–52], and it is obvious from these studies that the larvae are effective in localizing and infesting wood. But how do the tiny larvae locate point sources of ephemeral settling substrate in a vast ocean? It has long been hypothesized that shipworm larvae can sense and respond to chemical cues derived from wood [31,36,53], although this hypothesis has never been explicitly tested under ecologically realistic conditions. We found, for the first time, that natural populations of teredinid pediveliger larvae can find wooden settling substrate through detection of waterborne chemical cues. Although the larvae were prevented from direct physical contact with the substrate, they were 9 times more abundant around net bags containing wooden panels compared to empty net bags.

To our knowledge, few previously published studies have investigated responses of shipworm larvae to cues from wood [36,48,53,54]. Culliney [36] noted that swimming shipworm larvae (*T*. *navalis* and *Bankia gouldi*) in culture settled and started crawling when a piece of wood was placed in the culture, a behavior that was never seen in cultures without wood. Harington [53] exposed shipworm larvae (*Teredo* sp.) in a dish either to compounds extracted from "ordinary deal sawdust" or to malic acid administered through a capillary tube and found an attraction of larvae to the tube opening. However, there was no information on replication of statistical evaluation of the number of attracted larvae for any of these assays [36,53]. In a more ecologically relevant field experiment, Gara and co-workers [48] reported that larvae of *Bankia setacea* preferred to attack logs where the bark had been peeled back, possibly indicating a response to chemical cues released from fresh wood. Furthermore, fresh wooden samplers placed beside samplers that had been under attack from shipworm larvae for 8 weeks became significantly more attacked compared to fresh wooden samplers placed beside un-attacked samplers exposed to marine water for 4 weeks. The authors concluded that larvae were attracted to waterborne cues released from the previously attacked wooden material [48]. In contrast, Hoagland [54] found that pediveligers of *T*. *bartschi* preferred to settle on, or burrow in, wood soaked in artificial seawater for two weeks compared to wood previously held in the field for several months with or without adult shipworms. The combined results from the present and previous [36,48,53,54] studies clearly show that shipworm larvae can sense and respond to chemical cues released from submerged wood and/or from conspecifics, although the chemical identity of the cue(s) is not yet known.

Chemical cues may also interact with physical or hydrodynamic factors to induce larval settlement [24,55,56]. For example, small scale turbulence and flow influence the larva-substrate encounter rate through affecting the dispersal of chemical cues in the water column, as well as the trajectories and swimming behavior of invertebrate larvae [16]. It is relatively easy to imagine how chemical cues released from benthic substrates covering large areas, such as reefs of corals, oysters, or mussels, or forests of macroalgae, can influence settling by invertebrate larvae. In these cases, the settling substrate is abundant and most likely releasing a relatively large amount of chemical cues. For example, chemical cues released from coral reefs interact with the hydrodynamic conditions to change the behavior of nudibranch larvae relatively far from the source (they stop swimming and sink when encountering filaments of chemical cues in the water column above reefs), which increases the probability of larval-substrate encounter [17,57]. However, species with very specific habitat requirements, such as obligate parasites or species associated to hydrothermal vents, or whale and wood falls, have evolved means to settle on substrates that are much less abundant and constitute point sources of chemical cues. In these cases, it is much harder to understand how tiny larvae in a vast ocean may remotely sense the (presumably) very localized concentration gradients of chemical cues released from point sources and respond in a way that increase the probability of substrate encounter.

In a series of laboratory experiments, we explored the potential for remote detection of chemical cues that could explain the accumulation of shipworm larvae around wooden panels observed in the field experiment. A critical aspect is how larval swimming ability compares to flow around and close to the potential settling substrate. Video recordings and extrapolation to the competent stage indicate that cilia of the velum lobes can propel shipworm larvae with about 2 mm s^-1^ which is at least an order of magnitude slower than ambient free-stream flow velocities at the experimental site. The flume study showed that only within the boundary layer or in wakes behind protruding structures may larvae be able to control their movement towards the substrate. The boundary layer around the experimental wooden panels is thin and the mean flow velocity exceeds larval swimming velocity about 5 mm away from the panel surface (Fig 5). Crimaldi et al. [58] suggested that invertebrate larvae need a sufficient attachment time when the local velocity is below some critical level to allow settlement. Even in fast turbulent flow there may be short periods (lulls) of lower local velocities near the substrate that allow settlement if attachment time is sufficiently short. To our knowledge there is no study of attachment time and critical flow velocity for any bivalve larva, and no details about how hydrodynamic conditions affect shipworm settlement are known. However, Reidenbach et al. [59] calculated hydrodynamic forces acting on nudibranch larvae settling on coral reefs. Nudibranch larvae resemble bivalve larvae in their swimming ability, their velum organ, and that they use their foot to make contact with the substrate. In wave-dominated flow with a free-stream velocity of 9 cm s^-1^ over the reef top they calculated that attachment time need to be as short as about 1 s to allow attachment [59]. In slower flow or in more unidirectional flow the critical attachment time may be longer. Although highly uncertain, the successful settlement in our field experiment exposed to wave-dominated flow with velocities generally above 1 cm s^-1^ suggests that critical attachment time is shorter than 60 s [59]. This should allow ample time for shipworm larvae to sample the substrate for wood-based chemical cues.

The significant accumulation of shipworm larvae around wooden panels compared to controls (Fig 2) poses the question if larvae may respond to waterborne cues from some distance and so increase their encounter rate with wooden substrates. Koehl et al. [60] concluded that behavioral responses to waterborne cues could significantly affect settling rate of a nudibranch onto reefs with its preferred coral prey. The nudibranch larvae ceased up-ward swimming in response to chemical cues released by the coral and the resulting gravitational sinking increased settling rate. A similar chemical induction of larval behavior was found for oyster larvae increasing their sinking rate in response to a conspecific chemical cue [61]. In environments with high abundance of sunken wood, e.g. outside river mouths, such a response could increase encounter of suitable settling sites. For isolated, benthic as well as floating wooden objects induced sinking is not likely to increase encounter of suitable substrates. The release of a dissolved chemical marker in our flume experiment suggests that the chemical boundary layer is at most a few cm around isolated wood panels, as used in the field study (Fig 4B). In turbulent flow the instantaneous concentration field of a cue will be complex with intermittent peaks and thin filaments, which offer limited information about the direction to the source. Considering this lack of directional information and the slow swimming velocity (2 mm s^-1^) it seems unlikely that shipworm larvae are able to directly navigate towards the substrate except close to the diffusive layer, which is only about 1 mm thick (Fig 3C). Although slow-swimming invertebrate larvae may not be able to navigate horizontal chemical concentration fields at a distance there is a possibility that a more general response could increase encounter rate. Zilman et al. [62] proposed a mechanism where larvae may increase the encounter rate with solid objects through the interaction between self-propelled swimming and the vorticity in a laminar boundary layer flow. For the dimensions of the wooden panels used in this study the model in Zilman et al. [62] predicts a 10-fold increase of encounter rate for swimming compared to passive larvae at moderate ambient flow (1–3 cm s^-1^). If swimming activity incurs costs, e.g. increased predation risk [63], an option may be to increase swimming velocity only within the envelope of a waterborne cue and thus enhance encounter rate with the substrate when swimming through the boundary layer. Although Chan et al. [64] recently showed that gastropod larvae significantly modify the swimming velocity through adjustment of velum extension there is yet no information about this mechanism in shipworm larvae or in response to a chemical cue.

In summary, we conclude that the scope for remote detection of a waterborne cue is limited and that the likely explanation for the higher abundance of shipworm larvae associated with the wooden panels is a response to a waterborne cue during or after attachment on or very near the substrate. The perception of a waterborne cue could guide the larva in its decision to remain attached and settle or to detach and continue swimming and drifting until the next encounter with a solid substrate. Such settling behavior including substrate encounter, test of suitability, and then decide to settle or leave is similar to settling behavior described for some soft-sediment invertebrate larvae [65].

## Supporting Information

S1 Dataset Underlying data. The raw data underlying the statistical analyses and the figures in the present study.
(XLSX)
